# A retrospective cohort study to quantify the contribution of health systems to child survival in Kenya: 1996–2014

**DOI:** 10.1038/srep44309

**Published:** 2017-03-14

**Authors:** Rebecca Anthopolos, Ryan Simmons, Wendy Prudhomme O’Meara

**Affiliations:** 1 Rice University, Houston TX, USA; 2 Duke Global Health Institute, Durham NC, USA; 3 Duke University, Durham, NC, USA.

## Abstract

Globally, the majority of childhood deaths in the post-neonatal period are caused by infections that can be effectively treated or prevented with inexpensive interventions delivered through even very basic health facilities. To understand the role of inadequate health systems on childhood mortality in Kenya, we assemble a large, retrospective cohort of children (born 1996–2013) and describe the health systems context of each child using health facility survey data representative of the province at the time of a child’s birth. We examine the relationship between survival beyond 59 months of age and geographic distribution of health facilities, quality of services, and cost of services. We find significant geographic heterogeneity in survival that can be partially explained by differences in distribution of health facilities and user fees. Higher per capita density of health facilities resulted in a 25% reduction in the risk of death (HRR = 0.73, 95% CI:0.58 to 0.91) and accounted for 30% of the between-province heterogeneity in survival. User fees for sick-child visits increased risk by 30% (HRR = 1.30, 95% CI:1.11 to 1.53). These results implicate health systems constraints in child mortality, quantify the contribution of specific domains of health services, and suggest priority areas for improvement to accelerate reductions in child mortality.

Fifty percent of all childhood deaths occur in sub-Saharan Africa^1^,^2^. In Kenya, one in every 20 children die before their fifth birthday and preventable infectious diseases such as pneumonia, malaria, and diarrhea, account for 61% of mortality after the neonatal period^3^,^4^. These causes of death are avoidable through affordable and effective interventions. Therefore, it is reasonable to speculate that access to these life-saving interventions may be limited by the health systems that deliver them.

Peripheral health facilities in Kenya provide basic treatment services for acute conditions as well as basic prevention services such as immunization, antenatal care, and distribution of household health commodities including water treatment products and insecticide-treated bednets. There are notable geographic disparities in the distribution of health facilities across the country^5^,^6^. For example, in former Nyanza province in western Kenya, an estimated 99% of the population lives within 5 km of a public health facility, but in the poorer communities in the Northeastern province, less than 30% of the population lives within 5 km of a health facility^7^.

The uneven geographic distribution of facilities likely contributes significantly to inequitable access to health services and its impact has been studied in several contexts. Although distance to a health facility is strongly associated with utilization^8^,^9^,^10^,^11^, it is not consistently associated with improved health outcomes and survival. The relationship between geographic proximity and mortality is particularly inconclusive^12^ and studies exploring the effect of distance to health services on childhood mortality offer conflicting results^13^,^14^,^15^,^16^,^17^,^18^,^19^. One possible explanation for these differences is the lack of attention to other dimensions of access to health services such as affordability and quality. If services or commodities are limited in the most proximate facility, then any relationship between distance and survival may be obscured.

Our objective is to examine the extent to which health services in Kenya may drive the geographic variation in mortality among children under 5 years of age. We expand the concept of access in our analysis to include geographic distribution of facilities as well as quality and cost of services. In a retrospective cohort design, we link two large, nationally representative datasets that describe child survival and health service provision over nearly two decades. We hypothesize that 1) three domains of health services-geographic distribution, quality, and cost – at least partially account for geographic variation in child survival and 2) more health facilities, higher quality, and lower cost are associated with improved survival.

## Methods

### Data sources

We obtained Demographic and Health Surveys (DHS) for Kenya occurring in 1998, 2003, 2008/2009, and 2014. DHS have been described extensively elsewhere^20^. Briefly, DHS surveys are administered at regular intervals to document household health, intervention coverage, and childhood mortality in a representative sample of the population. Households are selected in a two-stage random sampling process. Information is collected from the household head and from every woman in the household aged 15–49 years. Households are categorized from poorest to least poor based on quintiles of a wealth index calculated from household asset ownership. Infant and child mortality are estimated from complete birth histories from all eligible women which document the date of birth and (if applicable) date of death for every child born to a woman in a sampled household.

We obtained information on health services in Kenya from the Service Provision Assessment (SPA) for 1999, 2004 and 2010. SPA are health facility based surveys conducted in a representative sample of government-owned and private health facilities representing a wide range of service provision levels (e.g., hospitals, clinics, health centers and dispensaries). The sample is representative at the provincial level and is stratified by service provision level. For this analysis, facility information is characterized at the province level using standard sampling weights.

### Data linkage

We integrated information from the DHS and SPA into a single analysis dataset. We matched children from the birth history table in the DHS to an SPA survey based on a child’s year of birth. To approximate the health services environment for each child, we used births within a maximum of 3 years from the first and last SPAs in 1999 and 2010, thus including children born as far back as 1996 and as far forward as 2013. For children born between SPA years, we assigned the SPA from the survey year closest to birth year.

### Outcome definition

We defined our outcome as survival beyond 59 months among children who survived to their first birthday. The observation period for each child was the time from birth to death, the date of the fifth birthday, or the interview date of the DHS – depending on which came first. If the interview date occurred first, then a child’s observation period was considered censored.

### Exposures

Health services were characterized based on three domains – geographic distribution, quality and cost. We measured distribution using the ratios of all health facilities or government-owned facilities per 1,000 persons. For quality, we used the number of clinicians on staff (nurses, clinical officers, midwives, and doctors), the presence of a physician, and the proportion trained in Integrated Management of Childhood Illnesses (IMCI). The number of clinical staff per facility was averaged across the representative sample of facilities in a province. The proportion of facilities in a province with at least one doctor on staff and the proportion (per facility) of clinical staff who have been trained in IMCI are similarly averaged over a province. The proportion of facilities in a province that charge any fees for sick child visits, childhood immunization, and normal delivery represented healthcare costs. We applied standard sampling weights provided with each SPA dataset when calculating aggregated characteristics across provinces^21^. We assigned children to a tertile (low, medium and high) of each health services variable using the province-SPA year level distribution.

### Control variables

In this study, our objective was to estimate the association between health services and child survival beyond 59 months. We used previous research to identify risk factors for survival that may also be correlated with health services but that arguably do not lie along the causal pathway between health services and survival^22^,^23^,^24^,^25^. Therefore, the premise of our variable selection approach was to control only for potential confounders, while excluding covariates through which the health services – child survival association may be mediated. For example, we selected not to control for immunization status because although the variable is likely correlated with survival, the degree of immunization is plausibly the product of the health services environment, and in turn, may account (at least in part) for survival outcomes.

We conceived potential confounders of the association between child survival and health services to be child, maternal, and household-level characteristics available in the DHS that are related to social and economic status, including maternal education, marital status, and household wealth. These characteristics may act as confounders if there are systematic differences in socioeconomic status among provinces that also drive interactions with health services. We include an indicator for whether a child resides in an urban versus rural cluster, which may relate to both socioeconomic status and proximity to health services. In addition, we control for child sex, maternal age, and birth order.

To control for secular nationwide trends in child survival and health services, we included birth year entered linearly. The linear form was selected in two ways: first, we plotted survival as a smoothed function of birth year. Second, we compared regression models that sequentially added linear, quadratic, and cubic polynomial terms based on Akaike information criterion.

### Statistical analysis

Descriptive statistics of child, maternal, and household risk factors, along with the three domains of health services, were computed. We used Pearson’s chi squared test to evaluate whether each risk factor differed by tertiles of health services. Overall and within each province, we calculated the probability of survival beyond 59 months based on the Kaplan Meier product limit estimator. We used the log-rank test to determine whether survival distributions differed statistically among provinces. We conducted the corresponding assessment of survival beyond 59 months and tertiles of each health services variable.

We fit Cox proportional hazards multilevel models to quantify geographic heterogeneity in survival beyond 59 months and estimate its association with health services. To account for potential correlation among survival outcomes of children born to the same mother and in the same province, the multilevel models were fit with random intercepts on province and mother^26^. We assume that due to shared, unobserved risk factors, 1) children born to the same mother will have more similar survival outcomes than children of different mothers, and 2) children from the same province will have more similar outcomes than children from different provinces. The province term allows us to estimate province level heterogeneity in survival while correcting inference for correlation among outcomes in the same province. The maternal-level random intercept performs the analogous function for births to the same mother. As estimates of excess risk^27^,^28^, the random intercepts reflect sources of heterogeneity at the maternal or province levels unaccounted for by risk factors included in models. We assume the maternal-level and province-level random intercepts are normally distributed and are independent of each other.

Applying a sequential model-fitting algorithm, we examined geographic variation in child survival and its relationship with health services. We fit a null model to estimate overall province level variation in survival, net of maternal based sources of heterogeneity (Model 1). Second, we fit a model that controlled for all covariates except the health services variables (Model 2). Third, in separate models, we added to Model 2 each health service variable (Models 3–10). We used our sequential model fitting algorithm to estimate the extent of unobserved sources of province based variation in child survival. To compare province-level heterogeneity between models^29^, we calculated the percent change in variance between the original model and the index model. Based on Models 3–10, we made inferences about the association between child survival and health services. In model estimation, we included a weight to correct for inter-province differences in the number of children available for analysis, in part stemming from province level variation in the number of sampled households by DHS survey year. This weight is equal to the inverse of the number of children in a province in a year, scaled by the number of children per province if all provinces had an equal number of children.

In sensitivity analysis, we evaluated the importance of province-level heterogeneity by conducting deviance analysis that compared Models 3–10 to their corresponding model that did not include a province-level random intercept. For each model, we also tested the proportional hazard assumption by assessing the correlation between scaled Schoenfeld residuals for a given covariate and a pre-specified function time. In addition to tests for each model covariate, we conducted a global test of all residual by time interactions.

All statistical analysis was conducted in statistical software R version 3.3.0 (The R Foundation for Statistical Computing, 2016).

## Results

The DHS in 1998, 2003, 2008/2009, and 2014 included 151,550 children. All eight provinces had an SPA conducted in 1999, 2004, and 2010 except for Northeastern which was missing information for 1999. After excluding children born more than 3 years before the first SPA in their province, and including only those who reached their first birthday, our study sample included 81,106 children born to 31,793 mothers. There were 1,494 deaths amongst these children.

Demographic information about the sample is presented in Table 1. Half of children were male, and almost three-quarters resided in rural areas (71%). The majority of mothers was married (79%) and had at least some primary education (86%). The proportion of children in each level of health services is shown in Appendix Table A.

### Geographic heterogeneity in child survival beyond 59 months and health services

**Figure 1:**
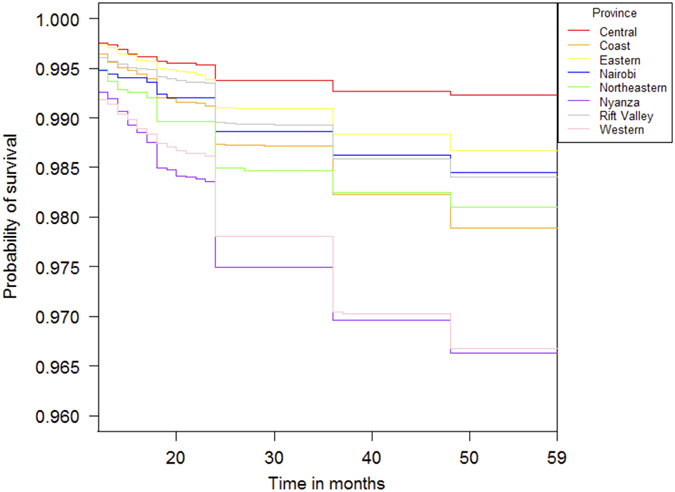
Kaplan Meier survival curves among children 12 months of age in Kenya by province, Kenya DHS during 1998–2014.

Figure 1 presents the Kaplan-Meier estimated survival distributions from 12 months onwards by province. Central province exhibited the highest survival rate (99.2%), while the lowest rates appeared in Nyanza (96.6%) and Western (96.7%). Survival distributions differed statistically by province (log-rank test, P < 0.0001).

**Figure 2:**
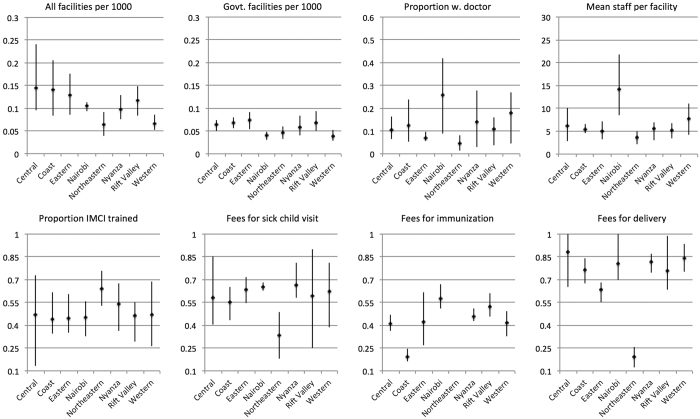
Mean and range of eight variables representing three domains of health services available to Kenyan families. No health services data is available from Northeastern in 1999.

A total of 1,523 facilities across the eight provinces were assessed. Health facilities per capita (geographic distribution) averaged across the three SPA surveys varied considerably between provinces, ranging from 1 facility per 4,150 people to 1 per 25,600 people (Fig. 2). In most provinces, government facilities composed about half of all facilities, but the proportion was lower in Nairobi (38%) and much higher in Northeastern (75%). The number of facilities increased over time, but the increase was largely due to the private sector, with the exception of Northeastern and Nyanza (Appendix Fig. 1). Only 5% of facilities in Northeastern had a doctor on staff compared to more than 25% in Nairobi. The mean number of clinical staff members per facility ranged between 5–8, with Northeastern and Nairobi again representing the two extremes. In contrast, a much larger proportion of clinical providers in Northeastern were trained in IMCI guidelines reflecting both the high proportion of government facilities as well as targeting of the program to underserved populations with high child mortality. Not all facilities charged user fees for child health and delivery services. Half to two-thirds of facilities charged fees for a sick child visit, including registration, consultation, laboratory services and drugs. Fees for individual facilities ranged from 0 to 20,400 Kenya shillings (median: 50 shillings). A slightly lower proportion of facilities charged fees for immunization and a larger proportion of facilities charged for delivery, ranging from 65% to 88%. Only 2% of facilities in Northeastern charged for immunization and only 19% charged for delivery.

In multilevel survival analysis, based on the null model (Model 1) overall province-level variation (i.e., variance of the random intercepts) in child survival was estimated to be 0.23 (Table 2). In the multilevel Cox survival model, the random effects are interpretable as measures of excess risk^27^,^28^. Using the standard deviation of the random effects, we can exponentiate to obtain an interpretation of risk relative to the average risk in the study sample. For example, the variance of 0.23 implies a standard deviation of 0.48 (i.e., 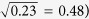 and 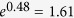. This indicates that children in a province 1 standard deviation above the norm have 60% higher risk of death. The estimated random intercepts themselves can also be interpreted in this way. The estimated random intercepts per province show that relative to average risk in the study, children in Western and Nyanza provinces were particularly worse off (HRR for Western = *e*^0.56^ = 1.75; HRR for Nyanza = *e*^0.70^ = 2.01), while residence in Central or Nairobi conferred a survival advantage (HRR for Central = *e*^−0.69^ = 0.50; HRR for Nairobi = *e*^−0.67^ = 0.51). Children in Nyanza faced double the average risk of death, while the risk of death among children in Central and Nairobi was halved. Large standard errors characterized the estimated random intercepts in other provinces.

Controlling for child, mother, and household level risk factors in Model 2 accounted for a small portion of the province-based variation. The variance of the province-level random intercepts decreased by about 20 percent from 0.23 to 0.18. Therefore, after adjusting for child, mother, and household level variables, children in a province 1 standard deviation above the average risk have about 50% higher risk of death 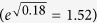. Estimated random intercepts shrunk towards the null but still represented sizable magnitudes. The HRRs for Western and Nyanza diminished to 1.52 and 1.79, respectively. The protective effect of Nairobi also became smaller (HRR = 0.71).

**Figure 3:**
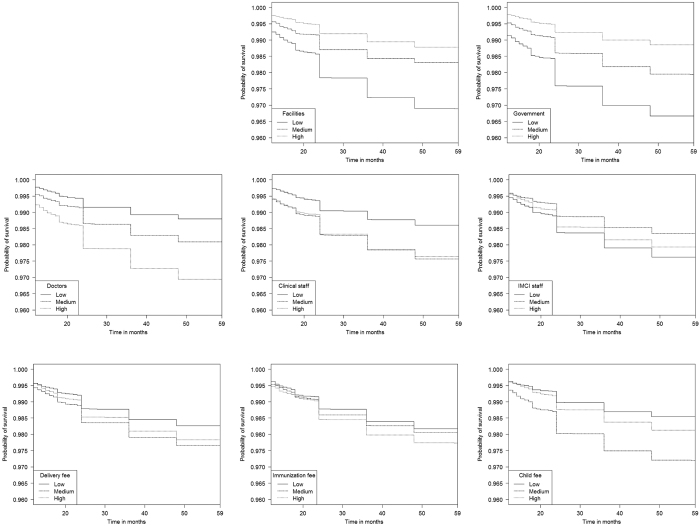
Map of Kenya showing the mortality hazard rate ratio per province for (**A**) Model 1 – Null model, (**B**) Model 2 – Individual and child-level covariates, (**C**) Model 3 – Model 2 plus all health facilities and (**D**) Model 8 – Model 2 plus proportion of facilities charging fees for sick child services. Maps were created in GeoDa 1.6.6^49^.

In Models 3–10, we separately added each health services variable to Model 2. Controlling for all facilities yielded the largest change in between-province variation in survival (Fig. 3). The estimated province-level variation dropped by over one-quarter to 0.13, which on the standard deviation scale, corresponds to a 40 percent increase in risk of death among children in a province 1 standard deviation above the norm. The HRR of Central became less protective by approximately 25 percent on the log scale, suggesting that some of the unobserved survival benefit is attributable to health facilities. The inclusion of fee for sick child visit also similarly reduced the province level unobserved heterogeneity, while delivery fee increased the estimated residual variance relative to Model 2 (province level variance = 0.23).

### Relationship between survival beyond 59 months, health services, and control variables

**Figure 4:**
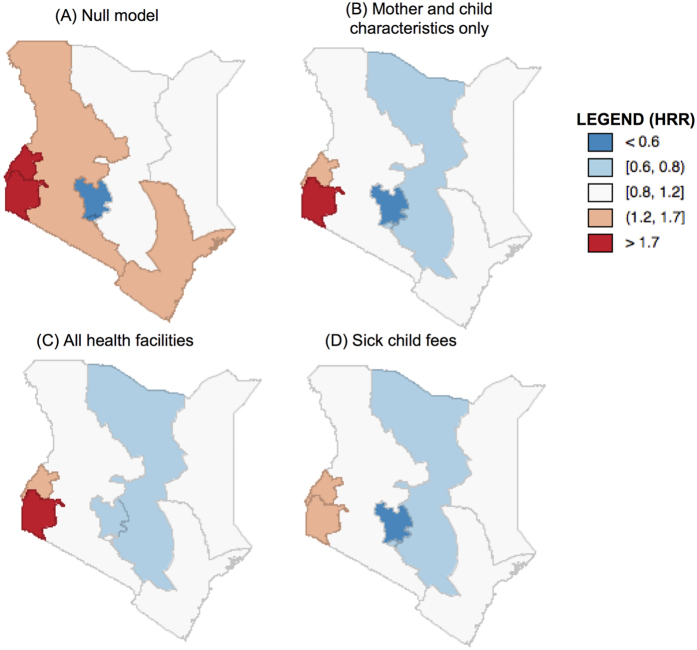
Kaplan Meier survival curves among children 12 months of age across tertiles of health services variables, Kenya DHS during 1998–2014 linked to SPA in 1999, 2004, and 2010.

Kaplan-Meier estimated survival curves by level of health services variables are shown in Fig. 4. According to log rank tests, survival distributions significantly differed by levels of health services (P < 0.01). The survival rate increased in a dose response fashion from low (tertile 1) to high levels (tertile 3) of government facilities (survival rate in low = 0.969, medium = 0.981, high = 0.990). However, the domain of quality exhibited unexpected relationships. Children in a province with a high proportion of facilities with doctors had a survival rate of 0.969, whereas the survival rate among children in a province with a low proportion of facilities with doctors was 0.988. For the health services variables related to cost, a low level showed the highest survival rate.

In Models 3–10 (Table 3), we separately added each health service variable to Model 2. After adjusting for potential confounders, facility density is only associated with child survival when private facilities are included. Children in high versus low levels of all facilities experienced a 28% decrease in risk of death (HRR = 0.72, 95% CI 0.59 to 0.89). A similar reduction in mortality was observed in the medium level. In contrast, all of the measures of quality of care were not associated with child survival, with the exception of an elevated risk of death associated only with the medium level of IMCI-trained staff. Children in provinces with higher prevalence of user fees (medium or high) for routine sick child visits had 30% higher risk of death than those living in provinces with lower prevalence of fees (e.g., High HRR = 1.3, 95% CI 1.11 to 1.53). For basic childhood immunization, children exposed to the highest prevalence of user fees had nearly 20% higher risk of death, although the 95% CI covered the null (HRR = 1.18, 95% CI 0.95–1.46). In contrast, fees charged for normal delivery appeared protective particularly when comparing medium to low levels of fees.

We observed expected associations with child, maternal, and household risk factors (Appendix Table B).

### Sensitivity analysis

The deviance analysis of Models 3–10 rejected the null hypothesis that the corresponding models without the random intercept were a better fit. We did not find evidence to reject the assumption of proportional hazards of the health services variables, with the exception of the indicator for high government facilities. The correlation coefficient between the scaled Schoenfeld residuals for high government facilities and transformed time was 0.07 (P-value < 0.01). A plot of the relationship showed that the slope was largely flat until the end of the follow-up period when an uptick occurred. For all models, the global hypothesis test of all interactions between the scaled Schoenfeld residuals and transformed time did not indicate violation of proportional hazards.

## Discussion

In this analysis, we explore the contribution of health services distribution, quality, and cost to child survival in Kenya over an 18-year period using health services data from three nationally representative surveys and a retrospective cohort of more than 80,000 children. We identify two major indicators of health services that have a large predicted impact on child survival, each of which account for a significant portion of the between-province heterogeneity in survival. First, we show that the number of health facilities per capita is correlated with substantial increases in survival, but only when private facilities are included in the estimate. Therefore, the growth of the private sector seems to be implicated in improved child survival. This may indicate better service provision, fewer drug shortages, or better staff performance in private facilities, although studies comparing quality of care in private and public facilities have not always demonstrated a difference between them^30^. This result could also be explained by growth of the private health sector in communities with greater ability to pay for health care, suggesting a complex relationship between local socioeconomic potential and survival.

Second, we see a detrimental effect of user fees levied for acute and preventive child services on child survival. Numerous previous reports have linked removal of user-fees to increased utilization^31^,^32^,^33^,^34^, however evidence of an impact on child mortality was inconclusive^35^,^36^,^37^. In our study, the impact of fees for sick child visits on mortality is remarkable and may reflect how difficult it is for families to absorb unplanned, potentially catastrophic, health expenses. By contrast, fees for scheduled immunizations are more modest and predictable. The small increase in child survival where delivery fees are prevalent may be related to the availability of higher levels of maternal care (i.e. emergency obstetric services), but how this relates to child survival (as opposed to neonatal survival) is unclear.

There is a surprising lack of association between survival and staffing of facilities. The number of staff and the presence of a physician are not correlated with survival. This may indicate that personnel are not the limiting factor in delivering adequate care, especially if faced with shortages of supplies or a population that cannot afford to access care. It may also suggest that the presence of highly trained physicians is not necessary to deliver many highly effective, simple interventions such as immunization and oral rehydration. The presence of IMCI-trained service providers also does not correlate with improved survival and intermediate-level of IMCI staffing was associated with higher mortality. This is not surprising given that IMCI training is often targeted to areas with very high child mortality^38^. It has also been documented that the effectiveness of IMCI can be severely hampered by lack of supplies^39^,^40^, and low health-worker morale^41^, all of which could confound the association between IMCI training and mortality in this analysis.

Although the relationship between individual health seeking behavior and health outcomes has been demonstrated^8^,^10^,^11^,^42^, a direct connection between health service delivery or health systems and health outcomes is underexplored in the literature. There are relatively few examples of studies that relate health systems factors to mortality in an analytical framework, and we are not aware of any that explore this relationship across an entire country. Previous studies of health services and child mortality fall primarily into two main groups; 1) studies which qualitatively compare trends in mortality to trends in health spending, scale-up of key interventions, or other development indicators^43^,^44^,^45^,^46^ or 2) studies that relate physical proximity and mortality^12^,^13^,^14^,^15^,^16^,^17^,^18^,^19^. The latter group has produced very mixed evidence about the effect of geographic access on morality. Other studies have documented intra-national heterogeneity in child mortality^22^,^47^,^48^, but they have failed to assign those differences to specific factors.

We evaluate the health services context over time and place using several important supply-side ‘domains’ which builds on previous studies that focus solely on geographic proximity to a facility^14^,^16^,^18^. We identify factors across multiple domains that are significantly correlated to child mortality. To our knowledge, this is the first time that these two rich data sources (DHS and SPA) have been linked to quantify the relationship between health services and outcomes. Our analysis shows that although the distribution of facilities is important, other health services characteristics are equally as important. Neglecting costs, for example, could contribute to the discrepancies in previous studies that only consider distance.

The current analysis has important limitations. First, the exposures variables had to be aggregated over large geographic areas, which neglects small-scale heterogeneity. As a result, the relationships revealed in this analysis may be an underestimate of their importance. Second, the measures of health services are imperfect although the method of data collection was consistent over time and space which allows for robust comparison. In particular, per capita facility density is an inadequate measure of geographic access given the variable geographic sizes of the provinces and the clustered nature of facilities within urban centers. It should therefore not be interpreted as measure of physical proximity, but rather representative of regional investment in health systems. Third, we estimate the health services context for a child by matching the nearest survey time-point to their date of birth. Finally, there remains significant heterogeneity in child survival between provinces after accounting for individual, maternal and health services factors, which could reflect unequal distribution of other resources between provinces, or differences in underlying health behaviors between communities.

Health systems are the foundational infrastructure through which life-saving interventions are delivered. However, their impact on population health is difficult to measure. Our analysis of large-scale survey data reveals important associations between health systems characteristics and child survival in Kenya, independent of utilization or specific interventions, and lends weight to the argument that user fees for child services are detrimental to child survival. Furthermore, the presence of highly trained staff do not appear to be the limiting factor in Kenya. Instead, more attention should be given to understanding the role of the private sector and investment in improving the distribution of health services infrastructure.

## Additional Information

**How to cite this article**: Anthopolos, R. *et al*. A retrospective cohort study to quantify the contribution of health systems to child survival in Kenya: 1996–2014. *Sci. Rep.* **7**, 44309; doi: 10.1038/srep44309 (2017).

**Publisher's note:** Springer Nature remains neutral with regard to jurisdictional claims in published maps and institutional affiliations.

## Supplementary Material

Supplementary Information

## Figures and Tables

**Table 1:** Descriptive statistics of analysis sample.

Child characteristics		N (%)
Birth order	Male	40916 (50)
	1	20188 (25)
	2–4	40997 (51)
	>4	19921 (25)
Birth year	1996–1999	17406 (21)
	2000–2003	19307 (24)
	2004–2007	20851 (26)
	2008–2011	16942 (21)
	2012–2013	6600 (8)
Mother & household characteristics		
Mother’s age	<20	13559 (17)
	20–24	25826 (32)
	25–29	20303 (25)
	30–34	13548 (17)
	>34	7870 (10)
Mother’s education	None or some primary	42782 (53)
	Finished primary	26873 (33)
	Finished secondary or higher	11451 (14)
Mother’s marital status	Married	63889 (79)
Wealth quintile	Poorest	25115 (31)
	Poorer	16843 (21)
	Middle	14851 (18)
	Richer	13014 (16)
	Richest	11283 (14)
Province	Nairobi	2671 (3)
	Central	6763 (8)
	Coast	10519 (13)
	Eastern	12528 (15)
	Nyanza	13136 (16)
	Rift Valley	22480 (28)
	Western	7974 (10)
	Northeastern	5035 (6)
Rural or urban	Urban	23153 (29)
Total		N = 81106

**Table 2:**
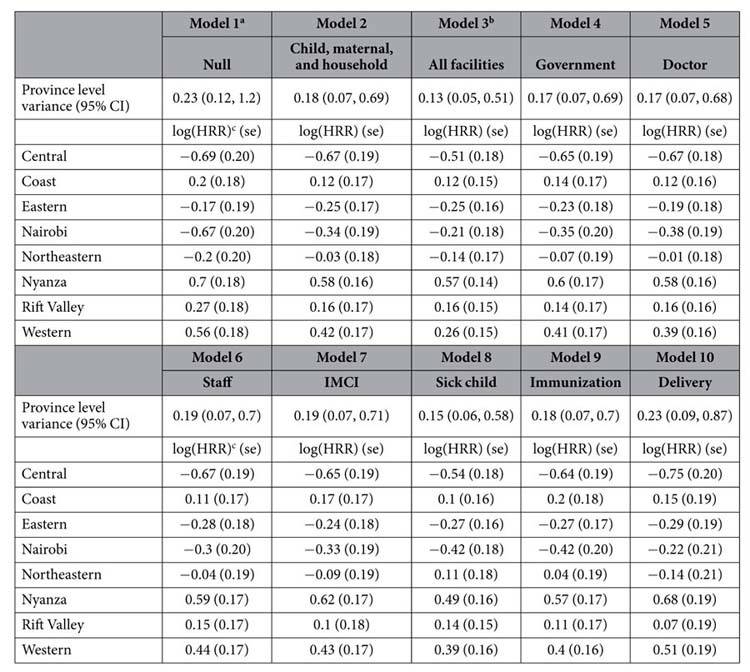
Province-level estimated residual heterogeneity and random intercepts from multilevel Cox proportional hazards models of child survival beyond 59 months.

^a^All models included a maternal level random intercept.

^b^Models 3–10 add each health services variable to Model 2 that included all control variables.

^c^The HRR are obtained by exponentiating the estimated random intercept for each province.

**Table 3:**
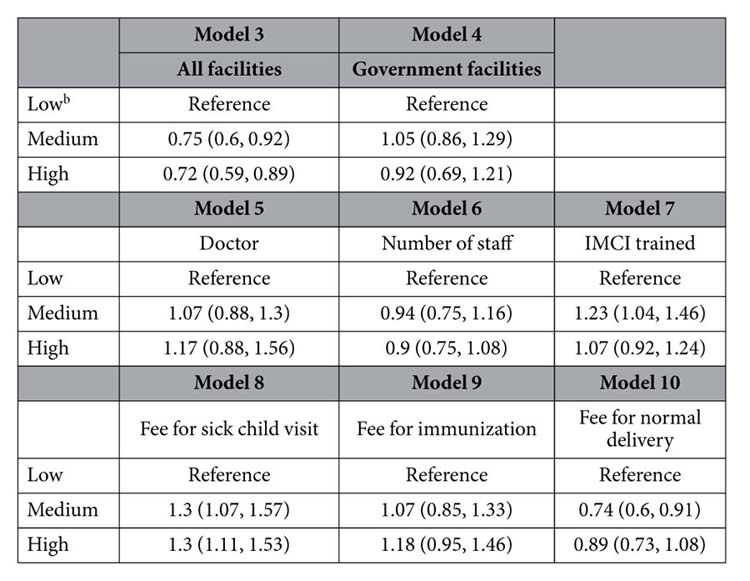
Multilevel Cox proportional hazards model of survival beyond 59 months among children who survived to their first birthday, Kenya DHS during 1998–2014 linked to SPA in 1999, 2004, and 2010^a^.

^a^All models were adjusted for child, maternal, and household level risk factors.
