# Supplementary material for: A retrospective cohort study to quantify the contribution of health systems to child survival in Kenya: 1996–2014
Source: Sci Rep. 2017 Mar 14;7:44309. doi: 10.1038/srep44309 (PMC5349518; doi:10.1038/srep44309)

A retrospective cohort study to quantify the contribution of health systems to child survival in Kenya: 1996-2014

Rebecca Anthopolos[1], Ryan Simmons[2], Wendy Prudhomme O'Meara* [2,3]

1. Rice University, Houston TX
2. Duke Global Health Institute, Durham NC
3. Duke University, Durham, NC

* corresponding author

Wendy Prudhomme O'Meara

Box 90519

Durham, NC

wpo@duke.edu

Appendix Table A: Number of children falling into each tertile of health systems variables

	Low	Medium	High
	N (%)	N (%)	N (%)
Facilities per 1000	30338 (37)	13948 (17)	36820 (45)
Government facilities per 1000	19462 (24)	29535 (36)	32109 (40)
Proportion of facilities with a doctor	32088 (40)	23494 (29)	25524 (31)
Mean clinical staff per facility	32614 (40)	30716 (38)	17776 (22)
Proportion of staff IMCI-trained per facility	24956 (31)	25225 (31)	30925 (38)
For sick child visits	25990 (32)	25653 (32)	29463 (36)
For immunization	23760 (29)	27647 (34)	29699 (37)
For normal delivery	33609 (41)	19019 (23)	28478 (35)

Appendix Table B: Multilevel Cox proportional hazards model of survival beyond 59 months among children who survived to their first birthday, child, maternal, and household risk factors, Kenya DHS during 1998-2014 linked to SPA in 1999, 2004, and 2010[a]

	HRR (95% CI)
Male	1.02 (0.92, 1.14)
Maternal age[a]	0.77 (0.68, 0.87)
Birth order	
1	Reference
2-4	1.07 (0.92, 1.24)
> 4	1.63 (1.3, 2.03)
Educational attainment	
None or some primary	Reference
Finished primary	0.81 (0.71, 0.93)
Finished secondary or higher	0.59 (0.47, 0.74)
Married	0.7 (0.61, 0.79)
Wealth quintile	
Poorest	Reference
Poorer	1.31 (1.11, 1.54)
Middle	1.1 (0.92, 1.32)
Richer	0.98 (0.8, 1.21)
Richest	0.9 (0.7, 1.17)
Urban	0.98 (0.84, 1.15)
Birth year[b]	0.92 (0.91, 0.93)

[a]Maternal age was centered at average age and scaled to represent a change from the 25th to the 75th percentiles of the distribution.
[b]Birth year was centered at 1996, the earliest year.

Appendix Figure 1: Changes in government and private health facility distribution by province in each SPA survey year.

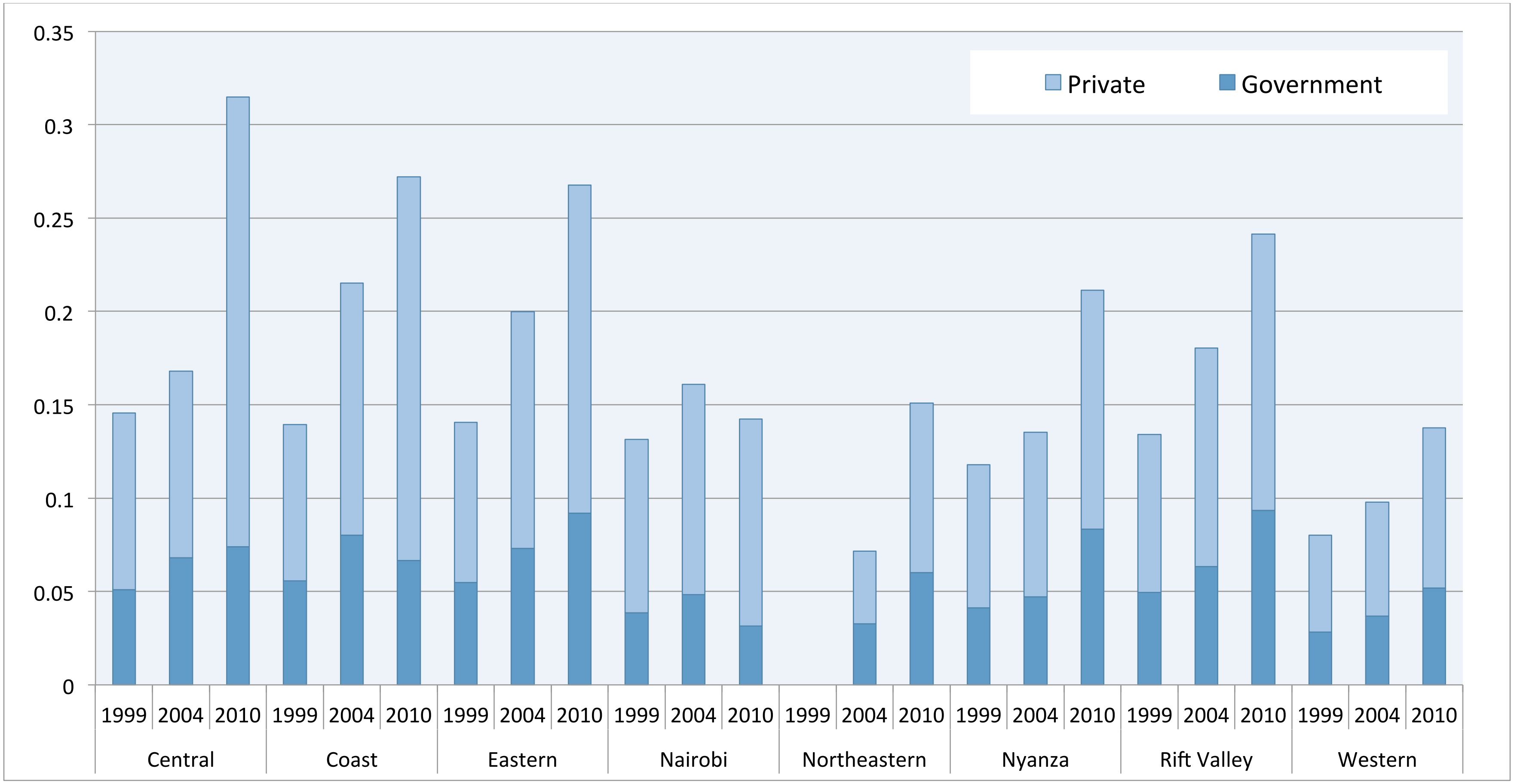

Supplement: Supplementary Information [file srep44309-s1.pdf]
